# Chronic postoperative complications and donor site morbidity after sural nerve autograft harvest or biopsy

**DOI:** 10.1002/micr.30588

**Published:** 2020-04-10

**Authors:** Ivica Ducic, Joshua Yoon, Gregory Buncke

**Affiliations:** ^1^ Washington Nerve Institute McLean Virginia USA; ^2^ Department of Surgery The George Washington University Washington District of Columbia USA; ^3^ The Buncke Clinic San Francisco California USA

## Abstract

**Background:**

The sural nerve is the most frequently harvested nerve autograft and is most often biopsied in the workup of peripheral neuropathy. While the complication types associated with these two procedures are well known, their clinical significance is poorly understood and there is a paucity of data regarding the complication rates.

**Methods:**

Pubmed search identified studies regarding complications after sural nerve harvest and biopsy. The data was grouped into sensory deficits, chronic pain, sensory symptoms, wound infections, wound complications, other postoperative complications, and complications impacting daily life. The incidence of each complication was calculated, and a chi‐square analysis was performed to determine if there were any differences between nerve biopsies and graft harvest with respect to each complication.

**Results:**

Twelve studies yielded 478 sural nerve procedures. Sensory deficits occurred at a rate of 92.9%, chronic pain at 19.7%, sensory symptoms at 41.1%, wound infections at 5.7%, noninfectious wound complications at 7.8%, and impact on daily life at 5.0%. The differences in wound infections, sensory symptoms, and impact on daily life between biopsies versus graft excisions were found to reach statistical significance (*p* < .05).

**Conclusions:**

Sural nerve excisions can cause chronic postoperative donor‐site complications. Given these complications, alternative available mediums for nerve reconstruction should be explored and utilized wherever appropriate. If an alternative medium is unavailable and nerve autograft must be harvested for nerve reconstruction, then patients should be counseled about risks for developing donor site complications that may negatively affect quality of life.

## 1 INTRODUCTION

Autogenous nerve grafts have been considered the gold standard for peripheral nerve gap reconstruction where tension free primary repair cannot be performed. Utilizing nerve tissue provides a conduit that allows for guided neurotrophic regrowth and utilizing host tissue eliminates the risk of rejection. Commonly used autografts include the sural nerve, medial and lateral antebrachial cutaneous nerves, superficial branch of the radial nerve, dorsal cutaneous branch of the ulnar nerve, superficial and deep peroneal nerves, intercostal nerves, and the posterior and lateral femoral cutaneous nerves (Ray & Mackinnon, 2010; Schenck, Lin, Stewart, et al., 2016).

The sural nerve is the most frequently harvested autogenous nerve graft for peripheral nerve reconstruction (Hankin, Jaeger, & Beddings, 1985; Mackinnon, 1989). When harvested, it offers substantial length for nerve gap bridging, is reliably found in the same anatomic location with minor variance, and is entirely sensory except for unmyelinated autonomic fibers (De Moura & Gilbert, 1984; Ortiguela, Wood, & Cahill, 1987). It is also frequently biopsied in the evaluation and workup of peripheral neuropathy for a variety of diseases (Argoc, Steiner, & Soffer, 1989; Wees, Sunwoo, & Oh, 1981). Although much has been written on the outcomes of the variety of nerve reconstructions utilizing the sural nerve, there is an equally significant paucity of data regarding the complications associated with sural nerve harvesting. The complications types following sural nerve excision and biopsy are well known; however, the rates and clinical significance of the complications are nebulous and poorly understood. Thus, the aim of this review is to provide insight into the clinical impact of these complications.

## 2 METHODS

### 2.1 Study inclusion

This review was guided by the Preferred Reporting Items for Systematic Review and Meta‐Analyses (PRISMA) checklist. A Pubmed literature review was performed to identify currently available studies regarding postoperative complications after sural nerve harvest and biopsy. There were no limits placed on study publication date, publication status, minimum follow‐up time, or design. All currently available studies including case reports were considered for inclusion. Relevant studies were identified and chosen by utilizing the search terms: sural nerve excision, sural nerve biopsy, sural nerve harvest, clinical complications, autograft, autogenous graft, sensory deficit, postoperative complaints, and morbidity. Studies were excluded if the article was not available in the English language. From the search results, the authors screened the titles or abstracts to determine relevance and study eligibility. The eligible studies were then reviewed independently by the authors and were included in the review upon reaching a unanimous consensus.

### 2.2 Data grouping

The data from the included studies were compiled and grouped into the following categories: sensory deficits, chronic pain, sensory symptoms, wound infections, wound complications, other postoperative complications, and complications impacting daily life. The sensory deficits were classified in a binomial fashion as either normal or decreased sensation including numbness compared to baseline pre‐operative sensation. Chronic pain is defined as pain or allodynia that has persisted to the follow‐up interview. Sensory symptoms include any one of the following symptoms: tingling, cold intolerance, paresthesia, dysesthesia, or irritating sensations. Wound infections were grouped separately from wound complications, which include any of the following symptoms: hypertrophic scar, poor cosmesis, or wound dehiscence. The other postoperative complications were postoperative hematomas and deep venous thromboses. Complications impacting daily life were determined by including patients who reported changes in their daily life or severe symptoms.

### 2.3 Data analysis

The incidence of each complication was then calculated separately for patients who underwent sural nerve biopsy versus sural nerve graft harvest and then calculated collectively utilizing the entire data set. For each complication, if the number or proportion of patients with the specific complication could not be determined from the study, then that study was excluded in the calculation of that specific complication. As the studies only provided the overall incidence of a complication and did not clarify how the complications were distributed among each patient, the greatest incidence of a single symptom within each complication category was utilized to determine the categorical complication rate for that study. A chi‐square analysis was also performed to determine if there were any significant differences between nerve biopsies and graft harvest with respect to each complication.

## 3 RESULTS

**FIGURE 1:**
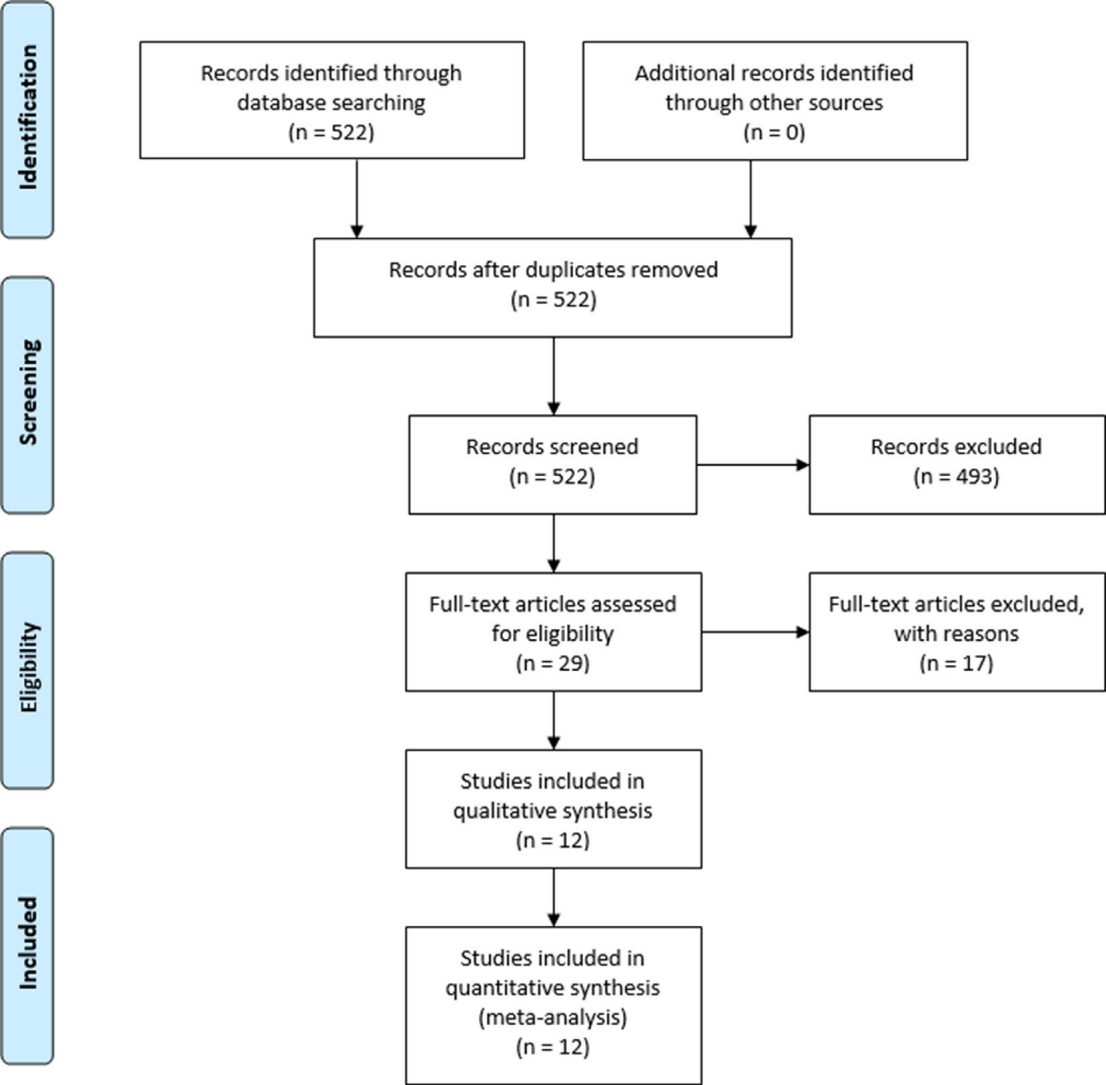
Study screening and selection algorithm

The literature search on PubMed conducted on April 4, 2019 identified 522 total studies. After screening of titles and abstracts, 493 studies were excluded. After reviewing the 29 remaining manuscripts, 17 were excluded due to clinical irrelevance to our primary question (Figure 1). A total of 12 studies fulfilled inclusion criteria yielding a total of 478 sural nerves of which 264 were biopsies and 214 were harvests as autogenous grafts in 471 patients (Catapano, Shafarenko, Ho, et al., 2018; Dahlin, Eriksson, & Sundkvist, 1997; Hallgren, Bjorkman, Chemnitz, & Dahlin, 2013; IJpma, Nicolai, & Meek, 2006; Kumar & Jacob, 2004; Lapid, Ho, Goia, & Clarke, 2007; Martins, Barbosa, Sigueira, et al., 2012; Miloro & Stoner, 2005; Perry & Bril, 1994; Rappaport, Valente, Hunter, et al., 1993; Staniforth & Fisher, 1978; Theriault, Dort, Sutherland, & Zochodne, 1998) (Tables 1 and 2).

**TABLE 1:** List of sural nerve graft harvest and biopsy studies with respective numbers of complications

Study	*n*	Follow‐up	Sensory deficits	Chronic pain	Sensory symptoms	Wound infections	Wound complications	Daily life impact
Lapid et al. (2007)	14	Greater than 5 years	9	0	1	x	x	0
Hallgren et al. (2013)	41	Greater than 14 months	38	21	12	0	0	7
Martins et al. (2012)	40	1 year	40	0	0	1	2	0
Staniforth and Fisher (1978)^a^	50	Between 3 months to 4 years with a mean of 18 months	50	19	20	1	5	0
Miloro and Stoner (2005)	26	Greater than 20 months with a mean of 36 months	26	0	6	x	0	4
IJpma et al. (2006)	29	Average of 26 years	22	5	10	x	7	5
Catapano et al. (2018)	14	Average of 1.8 years	13	4	3	0	0	1
Rappaport et al. (1993)	60	Greater than 6 months	n/a	3	x	6	7	0
Perry and Bril (1994)	106	Greater than 5.6 years	n/a	20	53	5	x	2
Theriault et al. (1998)	31	18 months	31	5	24	5	6	0
Kumar and Jacob (2004)	36	Between 3 to 10 months with a mean of 6.3 months	32	14	14	4	0	0
Dahlin et al. (1997)	31	Between 20 to 44 months	29	3	18	1	1	4

*Note:* x = outcome was not studied.

a Study in which two postoperative complications were incurred: calf hematoma and deep venous thrombosis.

**TABLE 2:** Rates of postoperative morbidity of sural nerve graft harvest versus sural nerve biopsy

	Sural nerve graft harvest (*n*/total)	Sural nerve graft harvest (% affected)	Sural nerve biopsy (*n*/total)	Sural nerve biopsy (% affected)	Total (% affected)
Sensory deficits	198/214	92.5%	92/98	93.9%	92.9%
Chronic pain	49/214	22.9%	45/264	17%	19.7%
Sensory symptoms^a^	61/214	28.5%	111/204	54.4%	41.1%
Wound infections^a^	2/143	1.4%	21/264	7.9%	5.7%
Wound complications	14/200	7.0%	14/158	8.9%	7.8%
Impact on daily life^a^	17/214	7.9%	7/264	2.7%	5.0%
Postoperative complications	2/214	0.9%	0/264	0.0%	0.4%

a Statistically significant differences between graft harvests versus biopsies.

### 3.1 Sensory deficits

Sensory deficits could be determined for 312 sural nerve interventions of which 98 were biopsies and 214 were harvests for graft. Ninety‐two of the biopsies and 198 of the graft harvests had sensory deficits, which resulted in sensory deficit rates of 93.8 and 92.5%, respectively and a collective rate of 92.9%.

### 3.2 Chronic pain

Chronic pain could be determined for all interventions. Forty‐five of the biopsies and 49 of the graft harvests had chronic pain, which resulted in chronic pain rates of 17 and 22.9%, respectively and a collective rate of 19.7%.

### 3.3 Sensory symptoms

Sensory symptoms could be determined for 418 sural nerve interventions of which 204 were biopsies and 214 were harvests for graft. One hundred and eleven of the biopsies and 61 of the graft harvests had sensory symptoms, which resulted in sensory symptom rates of 54.4 and 28.5%, respectively and a collective rate of 41.1%. The differences in sensory symptoms were found to be statistically significant (*p* < .05).

### 3.4 Wound infections

Wound infections could be determined for 409 sural nerve interventions of which 264 were biopsies and 145 were harvests for graft. Twenty‐one of the biopsies and 2 of the graft harvests had wound infections, which resulted in wound infection rates of 7.9 and 1.4%, respectively and a collective rate of 5.7%. The differences in wound infections were found to be statistically significant (*p* < .05).

### 3.5 Wound complications

Noninfectious wound complications could be determined for 358 sural nerve interventions of which 158 were biopsies and 200 were graft harvests. Fourteen of the biopsies and 14 of the graft harvests had noninfectious wound complications, which resulted in wound complication rates of 8.9 and 7.0%, respectively and a collective rate of 7.8%.

### 3.6 Impact on daily life

Impact on daily life could be determined for all interventions: 7 of the biopsies and 17 of the graft harvests had complications that negatively impacted daily life, which resulted in rates of 2.7 and 7.9%, respectively and a collective rate of 5.0%. The differences in the impact on daily life were found to be statistically significant (*p* < .05).

### 3.7 Other postoperative complications

**FIGURE 2:**
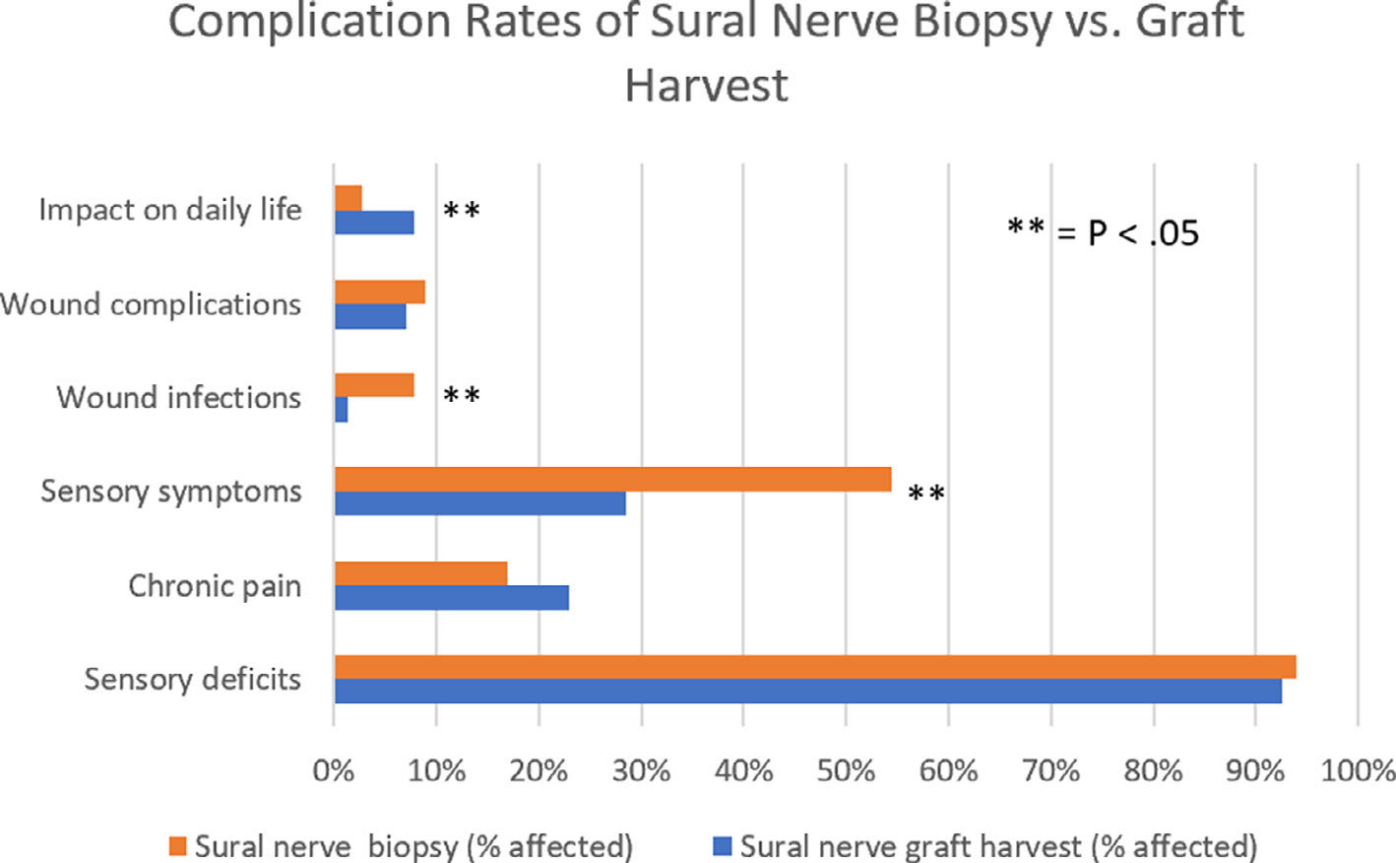
Comparisons of complication rates between sural nerve biopsy versus autograft harvest. Statistically significant differences found between impact on daily life, wound infections, and sensory symptoms (*p* < .05)

Postoperative hematoma and deep vein thrombosis for either procedure was reported with 0.4% incidence (Figure 2).

## 4 DISCUSSION

The most common and expected complication following sural nerve biopsy or autograft harvest is sensory deficit, which persisted in 92.9% of intervention sites. Intuitively, a loss of sensation in the distribution of the nerve will occur if the sensory nerve is resected irrespective of reason. However, the more salient point is to what degree will this area of sensory deficit recover over time. It has previously been established that the primary mechanism of sensorial recovery in these areas is collateral sprouting and not from proximal stump regrowth (Aszmann, Muse, & Dellon, 1996; Ehretsman, Novak, & Mackinnon, 1999). The studies documented such recovery; however, the extent of recovery was variable in both the proportion of patients and the degree of recovery.

Complete sensory recovery was rare and ranged from 0 to 11.1% of nerves in adults and 35% in children. Lapid and colleagues demonstrated that 35% of the graft harvest sites in children were able to reach normal sensation in comparison to controls who did not undergo any surgery (Lapid et al., 2007). In adults, the greatest rate of complete sensory recovery was shown by Kumar and colleagues to be only 11.1% among biopsy patients (Kumar & Jacob, 2004). Although only half the studies demonstrated complete recovery, all of the studies did find that there was some level of improvement in the sensory deficit. The determination of such improvements was heterogeneous, which ranged from subjective responses based on questionnaires and surveys to calculations of the deficient area with monofilament testing. Thus, a quantitative analysis of the degree of sensory recovery was not possible. Of the studies that provided objective data for the degree of sensory recovery, Martins noted that the mean area of reduction at 12‐month follow‐up after graft harvest was 54.2% whereas Theirault noted that the mean reduction at 18‐month follow‐up after biopsy was 91% (Martins et al., 2012; Theriault et al., 1998). The reason for the variability of the degree of sensory recovery could not be effectively parsed out due to the aforementioned heterogeneity of the data. However, we believe that with larger segments of nerve resection, the chronic area of sensory deficit after collateralization will be larger and would explain the difference in the degree of sensory recoveries between biopsies and graft harvests.

Staniforth and colleagues found that 16% of their patients developed neuromas and 44% were uncomfortable with the resultant sensory deficit (Rappaport et al., 1993). Recently, neuromas have been shown to occur in 6–8% of patients following traumatic injury (Van der Avoort, Hovius, Selles, et al., 2013; Vlot, Wilkens, Chen, & Eberlin, 2018). Staniforth's neuroma rate was significantly higher and this may be attributable to differences in operative technique that previously may have not minimized neuroma formation. Although the other studies did not explicitly diagnose and mention rates of neuroma formation, given the myriad of sensory symptoms among the patient population, we suspect that there is a small but significant portion of the population with stump neuromas. This is concerning because if these neuromas become symptomatic, then they can be a significant source of chronic pain and negatively impact quality of life (Ashkar, Omeroglu, Halwani, et al., 2013; Lu, Sun, Wang, et al., 2018; Vernadakis, Koch, & Mackinnon, 2003). Several anatomical studies helped delineate the surgical anatomy of the sural nerve and its clinical implications (Coert & Dellon, 1994; Eastwood, Irgau, & Atkins, 1992; Williams, 1954). Further studies have suggested means to limiting donor site pain by ensuring the proximal stump does not lie within the gastrocnemius muscle and limiting neuroma formation by the inclusion of the medial and lateral sural nerves in the harvest (Coert & Dellon, 1994; De Moura & Gilbert, 1984; Ducic & West, 2009). Ideally, additional subgroup analyses of the operative techniques and their associated outcomes would be performed; however, we were limited by the availability of the operative techniques and sample size.

The other studies also did not show as great of rates of dissatisfaction; however, a small but significant portion of patients did endorse that their chronic symptoms did negatively impact their daily life. Hallgren reports that the daily activities of 7 patients were affected with 2 of those patients reporting a severe impact, Miloro reports 4 patients with affected activities of daily living, Theriault reports 24 patients with persistent irritating symptoms, Dahlin reports 4 patients with severe symptoms affecting their daily lives, and Perry found that 2 patients developed severe sensory symptoms (Hallgren et al., 2013; Kumar & Jacob, 2004; Miloro & Stoner, 2005; Perry & Bril, 1994; Theriault et al., 1998). The majority of patients did not have symptoms that were significantly impactful; however, 2.3% of biopsy patients and 6.4% of graft harvest patients did and that is not a negligible proportion of patients. This difference between biopsies and graft harvest was found to be statistically significant. It was difficult to determine how daily activities were specifically affected as there was no standardized measure or criteria that was utilized throughout the studies. Some authors such as Hallgren and Dahlin determined this outcome with subjective surveys while others such as Miloro and Perry utilized specific functions and activities such as ADLs (activities of daily living), routine processes such as sleep, or the need for medications. Given this wide spectrum, we believe that there is higher proportion of patients who have a noticeable negative impact on their quality of life, which is not fully reflected in this data set; however, the perceived clinical impact on the quality of the life by the patient is undeniable. We believe that the length of nerve that is excised is responsible for this difference; however, due to the unavailability of such data points for analysis, we were unable to explore farther.

In patients who underwent nerve autograft harvest for nerve reconstruction, nearly all the patients were satisfied and would elect to undergo the procedure again given their targeted functional outcomes. However, in the patients who were biopsied, a significantly smaller portion of patients would elect to be rebiopsied. Dahiln found that only 10% of diabetic patients who were biopsied would undergo the biopsy again whereas Perry found that 59% of all patients would undergo a biopsy again (Dahlin et al., 1997; Perry & Bril, 1994). The reasons for such hesitance and dissatisfaction are multifactorial, but a major component is likely the fact that some of the biopsies were nondiagnostic and resulted in chronic symptoms.

Wound infections were found to be higher among patients that underwent biopsies compared to graft harvest patients. We would have liked to pursue further subgroup analysis but given the heterogeneity and lack of patient data we were unable to do so. Theriault found that a higher proportion of diabetic patients undergoing nerve biopsy had wound infections in comparison to nondiabetics (Theriault et al., 1998). We suspect that underlying comorbidities and more specifically diabetes is the primary factor in the difference between the two groups, because diabetes is a well‐established risk factor for postoperative infections (Tan, Oh, & Kwek, 2015). The diabetic patients who were getting sural nerve biopsies for the workup of peripheral neuropathy at baseline likely had poorly controlled diabetes as indicated by the progression of neuropathy. Additionally, persistent sensory symptoms were found to be significantly more prevalent within the sural nerve biopsy group. We suspect that the underlying comorbidities that served as the impetus for the progression of peripheral neuropathy likely continued to persist if not progress.

The studies offered a sizeable collection of patients, but there were several limitations and differences between the studies. The follow‐up ranged from 3 months to greater than 5 years; however, most patients had follow up of at least 6 months. As mentioned before, the manner in which the patients were interviewed and the manner the data was collected was heterogeneous thus limiting any subgroup analysis or full characterization of the patient demographics of this review population. Additionally, the operative techniques were not clearly outlined for most of the studies. Among the studies, only Lapid and colleagues conducted a control matched study using separate patients while Catapano and colleagues utilized the contralateral limb as their controls. The rates of each complication or symptom were well reported; however, the lack of a standardized questionnaire and patient specific information made it difficult to perform any subgroup analysis.

These complication rates should be utilized in counseling the patient of the operative risks and also in guiding operative planning. In regard to sural nerve biopsies, the complications need to be weighed against the diagnostic yield, which was not discussed here. These patients should also be acutely aware of their increased potential for wound infections based on their comorbidities. In regard to graft harvest, the current literature suggests that there are alternative nerve reconstruction mediums that do not require autogenous harvesting with comparable results (Mauch, Bae, Shubinets, et al., 2019; Safa & Buncke, 2016). Thus it is our opinion that those reconstructive options should be considered first, as donor site morbidity can be significant and life impairing. Furthermore, we would have liked to pursue a comprehensive review of all the complications associated with the various types of autografts; however, the paucity of the data in the literature prevented us from effectively doing so. We suspect that the types of complications in harvesting other nerve autografts would be similar.

## 5 CONCLUSIONS

Sural nerve excision for biopsies and autogenous grafts has the potential for persistent chronic postoperative morbidities. Nearly everyone has a residual sensory deficit that may improve to a variable extent over time due to collateral sprouting of nearby nerves. In addition, this metanalysis found that 19.7% of patients may have chronic pain, 5.7% may have wound infections, 41.1% may have some chronic nonpain related sensory symptom, 7.8% may have noninfection related wound complications, and 5.0% may have symptoms that noticeably affect their daily life. Given these postoperative morbidities that may chronically negatively affect quality of life, patients undergoing sural nerve intervention should be counseled of these significant risks. For patients needing nerve reconstruction, alternatives to sural or other autograft nerve harvest should be explored, when current alternative techniques are justified and supported by evidence‐based outcomes. Unlike anecdotal discussions about autograft harvest or nerve biopsy complication types and rates, this evidence‐based summary should serve as an objective tool for both surgeon and the patient when choosing a treatment module, aiming to improve patient safety and outcomes.

## CONFLICT OF INTEREST

The authors have no financial interest to declare in relation to the content of this article. Authors independently conducted and received no funding for the study. Additional affiliations: Dr. Ducic is Medical Director of Axogen; Dr. Buncke is consultant for Axogen and Polyganics; Dr. Yoon has no other affiliations.
